# Knowledge of nursing teachers about health promotion for the LGBTQIA+ population

**DOI:** 10.1590/1980-220X-REEUSP-2024-0178en

**Published:** 2024-11-29

**Authors:** Gesiany Miranda Farias, Jussara Gue Martini, Mara Ambrosina de Oliveira Vargas

**Affiliations:** 1 Universidade Federal de Santa Catarina, Departamento de Enfermagem, Florianópolis, SC, Brazil.

**Keywords:** Faculty, Nursing, Sexual and Gender Minorities, Teaching, Health, Docentes de Enfermería, Minorías Sexuales y de Género, Enseñanza, Salud, Docentes de Enfermagem, Minorias Sexuais e de Gênero, Ensino, Saúde

## Abstract

**Objective:**

To describe the knowledge of Nursing teachers about the LGBTQIA+ population’s health.

**Method:**

Qualitative, exploratory and descriptive research, whose data collection strategy was through a World Café. The study was carried out with nursing professors from a public higher education institution located in the northern region of Brazil. Data analysis was performed using the thematic or categorical approach. The software Atlas.ti® was used for coding and organization.

**Results:**

The development of the World Café involved the participation of 10 professors, from different curricular activities. The audios obtained during the World Café were thoroughly examined, and thematic categories were identified for the presentation of the results, namely: LGBTQIA+ Education, Training and Health; From Prejudice to Recognition of Rights; and Reflective Education in the Recognition and Deconstruction of Social Determinants.

**Conclusion:**

The analysis of the results indicates the importance of understanding discursive practices in the training process, considering prejudice and discrimination as social determinants that affect health promotion.

## RESUMO

**Objetivo:**

Descrever o saber de docentes de Enfermagem sobre a saúde da população LGBTQIA+.

**Método:**

Pesquisa de abordagem qualitativa, do tipo exploratória e descritiva, cuja estratégia de coleta de dados foi por meio de um World Café. O estudo foi realizado com docentes de enfermagem de uma instituição de ensino superior de natureza pública situada na região norte do Brasil. A análise dos dados foi realizada utilizando a abordagem temática ou categorial. Para a codificação e organização utilizou-se o software Atlas.ti®

**Resultados:**

O desenvolvimento do World Café contou com a participação de 10 professoras, provenientes de diversas atividades curriculares. Os áudios obtidos durante o World Café foram minuciosamente examinados, e categorias temáticas foram identificadas para a apresentação dos resultados, sendo elas: Ensino, Formação e Saúde LGBTQIA+; Do Preconceito ao Reconhecimento dos Direitos; e Educação Reflexiva no Reconhecimento e Desconstrução de Determinantes sociais.

**Conclusão:**

A análise dos resultados indica a importância de compreender as práticas discursivas no processo de formação, considerando o preconceito e a discriminação como determinantes sociais que afetam a promoção da saúde.

## RESUMEN

**Objetivo:**

Describir el conocimiento de los docentes de Enfermería sobre la salud de la población LGBTQIA+.

**Método:**

Investigación con enfoque cualitativo, exploratorio y descriptivo, cuya estrategia de recolección de datos fue a través de un World Café. El estudio se realizó con profesores de enfermería de una institución pública de educación superior ubicada en la región norte de Brasil. El análisis de los datos se realizó mediante el enfoque temático o categórico. Para la codificación y organización se utilizó el software Atlas.ti®.

**Resultados:**

El desarrollo del World Café contó con la participación de 10 docentes, de diversas actividades curriculares. Los audios obtenidos durante el World Café fueron examinados minuciosamente y se identificaron categorías temáticas para la presentación de los resultados, a saber: Educación, Formación y Salud LGBTQIA+; Del prejuicio al reconocimiento de derechos; y Educación Reflexiva en el Reconocimiento y Deconstrucción de Determinantes Sociales.

**Conclusión:**

El análisis de los resultados indica la importancia de comprender las prácticas discursivas en el proceso de formación, considerando el prejuicio y la discriminación como determinantes sociales que afectan la promoción de la salud.

## INTRODUCTION

Nursing students education must be guided by the field curricular guidelines and the principles of the Brazilian Public Health System (SUS), seeking critical and reflective training to develop the skills and competencies required to exercise the profession, focusing on health promotion for the Lesbian, Gay, Bisexual, Transgender, Queer, Intersex, Asexual (LGBTQIA+) population^(1)^. Thus, it is understood that the adoption of inclusive teaching practices in the learning context should be a norm, aiming to train nurses and future leaders sensitive to the needs of the population^(2)^.

Therefore, it is essential that the inclusion of topics related to sexual orientation and gender identity in nursing training and practice is promoted from the undergraduate period onwards. These themes are Social Determinants of Health (SDH) and highlight disparities in health indicators for the LGBTQIA+ population^(3)^.

National studies on nursing training point to insecurity and lack of skills in caring for the LGBTQIA+ community, highlighting the need to review course curricula for a more qualified approach^(4,5,6)^. The lack of adequate training to provide assistance to this group is a concern also highlighted in international studies. This gap can result in inadequate care, perpetuation of stereotypes, and even cause discomfort during care^(7,8)^.

Corroborating these statements, the *National League for Nursing* (NLN) and the *American Association of Colleges of Nursing* (AACN) highlight the need to incorporate the principles of diversity, equity, and inclusion (DEI) into nursing curricula^(2)^.

It is important to note that society is influenced by what we consider to be true, mainly through social institutions that validate and reinforce discourses. This is reflected in pedagogical practices and all their elements, such as books, editions, libraries, laboratories, researchers, and professors. In addition, Foucault analyzes how knowledge is presented to society, exploring the way it is valued, distributed and attributed, especially in pedagogical practices within institutions^(9)^.

Thus, it is believed that the education of health professionals has to comprise these social and historical constructs, as well as follow the changes taking place in society, having as one of its main guiding principles the health care needs of the population, aiming at the promotion of health, humanization, ethics, and social inclusion^(10,11)^.

Nurses play an essential role in health care; however, they have to understand how prejudice interferes with health care and promotion. This is essential to guarantee sensitive and respectful embracement, ensuring that everyone feels comfortable and respected during assistance^(12)^.

It should be noted that recognition and understanding of sexual and gender diversity provides nurses with the skills to offer more effective and inclusive care, thus contributing to the promotion of health and well-being of the entire community^(10)^.

In Brazil, there is an LGBT Health Policy, but there is a social and political movement to include other terms, such as intersex, queer, asexual, among others. This policy serves as a guiding document that legitimizes the needs and particularities of this group, reinforcing SUS’s commitment to the principles of universality, integrality, equity, and social participation^(13)^. In this regard, the objective of this study is to describe the knowledge of Nursing professors about the LGBTQIA+ population’s health.

## METHOD

### Design of Study

This study is a qualitative research of an exploratory and descriptive nature, whose data collection strategy was a World Café. This data collection technique was developed in the 1990s by Juanita Brown and David Isaacs and is used in participatory research. This methodology encourages collaboration in activities involving group interaction, allowing the sharing of knowledge and information^(14)^. To ensure the quality of the research report and the preparation of the manuscript, the translated and validated Portuguese version of the guide *Consolidated Criteria for Reporting Qualitative Research* (COREQ)^(15)^ was used.

### Study Setting and Participants

The study was carried out at a public higher education institution located in the northern region of Brazil. Inclusion criteria: permanent nursing faculty. Exclusion criteria: professors on vacation or some type of leave during the data collection period.

### Data Collection

The professors were invited to participate in the study by email and telephone. During this phase, the objective of the research and its justification were explained. To explore the information, a priori, a pilot study was used^(16)^, which is important for improving data collection in research. To carry out the World Café, the availability of days and times of the research participants was first considered, with the aim of scheduling a suitable location for data collection.

The World Café experience took place during a meeting in July 2023; however, the period between planning and holding the World Café was 6 months. The dynamic was carried out to bring together nursing professors from different curricular activities, in line with the guiding principles of this participatory method, with the stages described in Chart 1.

**Chart 1:** Guiding principles of the World Café – Belém, Pará, Brazil, 2023.

1. Context definition	The objective and invitations were described, as well as discussion topics that helped to develop the content and questions to be presented as guidelines.
2. Warm and hospitable environment	An invitation and a welcoming environment were established that could allow participants to be comfortable and safe to express their opinions and suggestions on the topics under discussion, with support materials such as tables, chairs and stationery items for the participants’ comfort and expression.
3. Explore questions relevant to the topic being worked on	At this stage, a triggering question was used for group discussion during the conversation round or to expand on the debates already established.
4. Encourage contributions from all participants:	Participants were encouraged to take part in the discussion, on the proposed theme, but not only that, they could contribute by writing ideas or any other pertinent statement.
5. Connection of diverse perspectives	It consisted of bringing together the various participating groups, providing an exchange of knowledge, enriching the debate and enabling new insights.
6. Listen to Patterns and Insights	It aims to better connect with the group through shared listening.
7. Share collective findings	Each group or its host (representative) should present the insights and knowledge discussed at each group change to all participants.

Source: Prepared by the author based on the text *Design Principles* published in *The World Café* ^(17)^.

The meeting included two rounds of questions that were answered collectively by each group, with an emphasis on the themes of sexuality and sexual diversity. Participants were divided into equally distributed groups, with a host selected to moderate each group. The time allotted for the dialogue and discussion round was 20 to 30 minutes, with a 5 to 10 minute break between each one. Both rounds took place on the same day. The discussions were audio recorded for later analysis.

Following each discussion cycle, participants transitioned to the next table, where the group host took responsibility for sharing the material and information gathered by previous participants. In addition to facilitating this transition, the host also played the role of an attentive listener to the reflections presented by the group, aiming at stimulating conversation around the proposed topic.

As we aimed to stimulate discussion in the context of the first group, the video shown on YouTube was used as a stimulator for dialogue: *¿Cuál es la diferencia?*, designed by Centros *Libres de Homofobia de Uruguay*, having as organization *Colectivo Ovejas Negras, Ministerio de Salud Pública, Administración de los Servicios de Salud del Estado Dirección, Universidad de la República Oriental del Uruguay and UNFPA Uruguay.* In the second group, an excerpt taken from the National Policy for Comprehensive Health for Lesbians, Gays, Bisexuals, Transvestites and Transsexuals was presented, aimed at encouraging and enriching the discussion^(13,14,15,16,17,18)^.

After the questions and discussions in the groups were concluded, the participants’ contributions regarding each of the issues discussed in the groups were presented. These interactions were recorded in audio, in addition to photographic records of the materials created by the groups. Each host shared their collective insights and discoveries at each group change; however, other participants were encouraged to share their own experiences and knowledge related to the questions proposed during the rounds of each group and after their conclusions. Numerical codes (01, 02, 03, 04 and 05) were used to present the speeches of the World Café participants, who decided to present their contributions at the end.

### Data Analysis

Data analysis was performed using the thematic approach. According to Minayo^(19)^, thematic content analysis approaches social reality through reflection on data interpretation. The analytical process was carried out in three stages: pre-analysis, material exploration, processing and interpretation of results. Furthermore, an approximation with Foucaultian references was used as theoretical support. Data were coded in the software Atlas.ti® and then categories were established for analysis.

### Ethical Aspects

The research was carried out after approval by the Research Ethics Committee, submitted to Plataforma Brasil, under opinion number 5.771.494 and with authorization through the Free and Informed Consent Form (FICF).

## RESULTS

**Figure 1:**
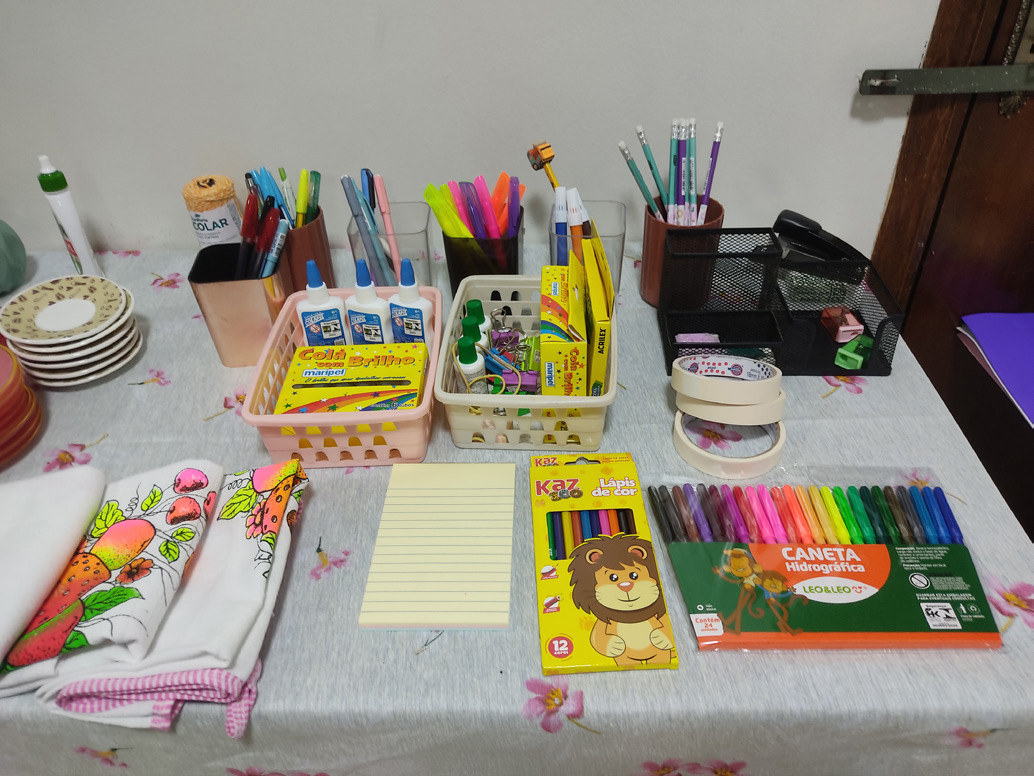
Stationery materials – World Café- Belém, Pará-Brazil, 2023.

**Figure 2:**
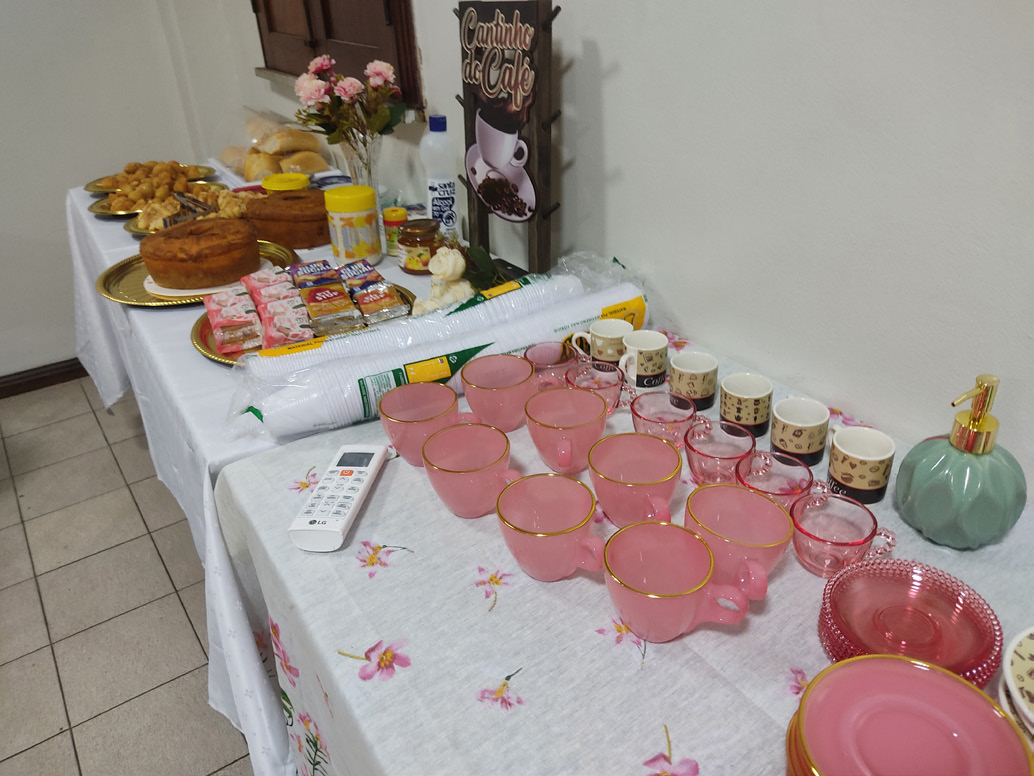
Coffee, tea, and snacks- World Café- Belém, Pará-Brazil, 2023.

The development of the World Café involved the participation of 10 professors, most of them women, from different curricular activities. For reasons of anonymity, curricular activities were not disclosed. At the beginning of the meeting, the participants were presented with the objective of the research and everyone was introduced to the space, which provided stationery materials (Figure 1) and coffee, tea and snacks (Figure 2).

Divided into two groups, the participants were able to choose their initial group, being distributed equally, with the hosts being chosen by the researcher, considering affinities among them. The groups were given the autonomy to designate representatives who would share their insights and perceptions. It should be noted that all participants were encouraged to contribute with their perspectives to the discussions.

The video *“¿Cuál es la diferencia?”* was highlighted for showing the presumption of heterosexuality as an obstacle in health care, fragmenting care and hindering the provision of adequate health guidance. After the video was shown, the first question was asked to the first group formed to begin the dialogue: How to promote education that embraces sexual diversity? Then, the other group began reading the text of the National Policy for Comprehensive Health for Lesbians, Gays, Bisexuals, Transvestites and Transsexuals, which preceded the presentation of the second question: How can we promote critical, reflective education focused on SUS and that understands LGBTA+phobia as a social determinant of health?

**Figure 3:**
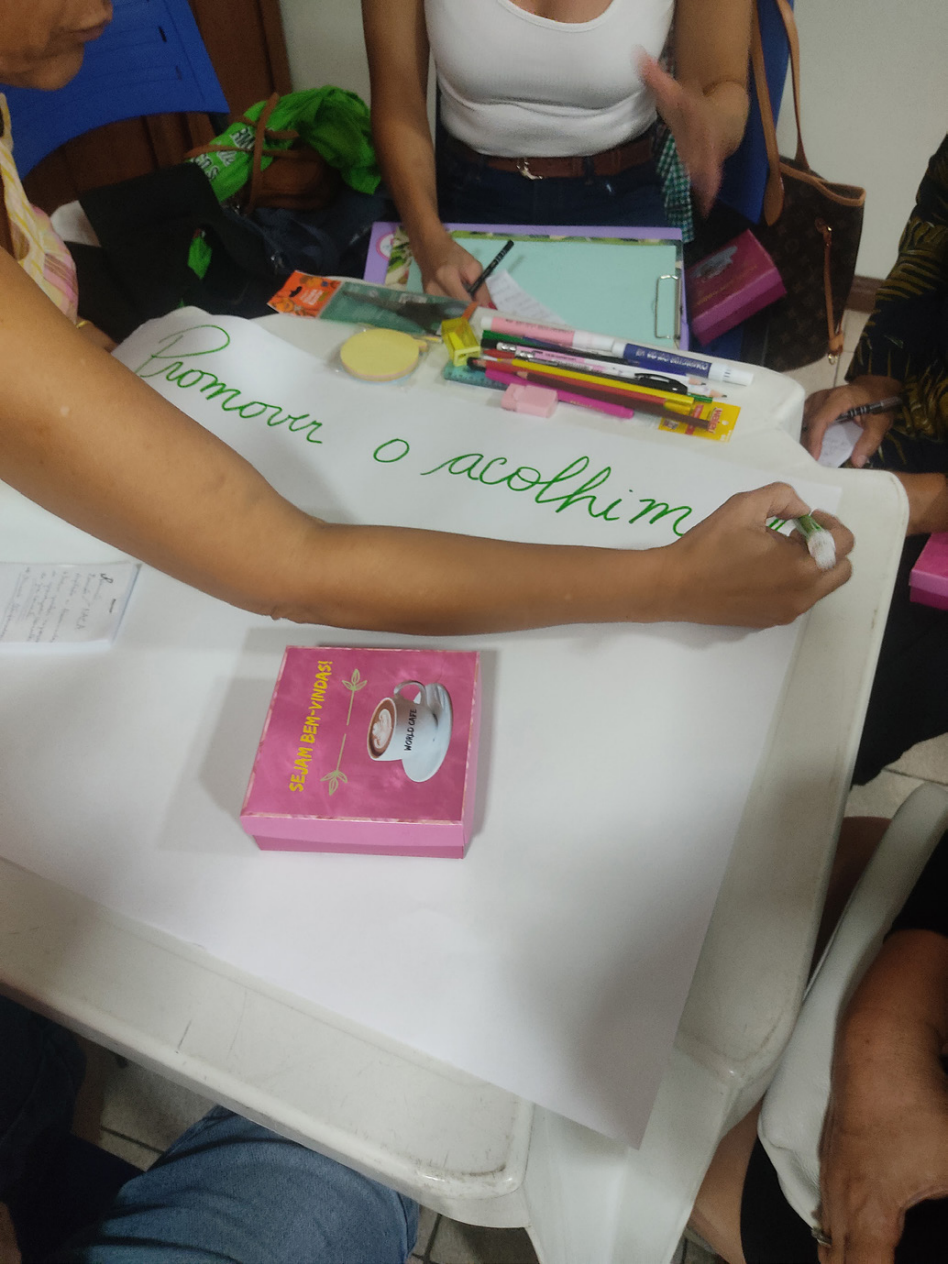
Production of insights- World Café- Belém, Pará-Brazil, 2023.

**Figure 4:**
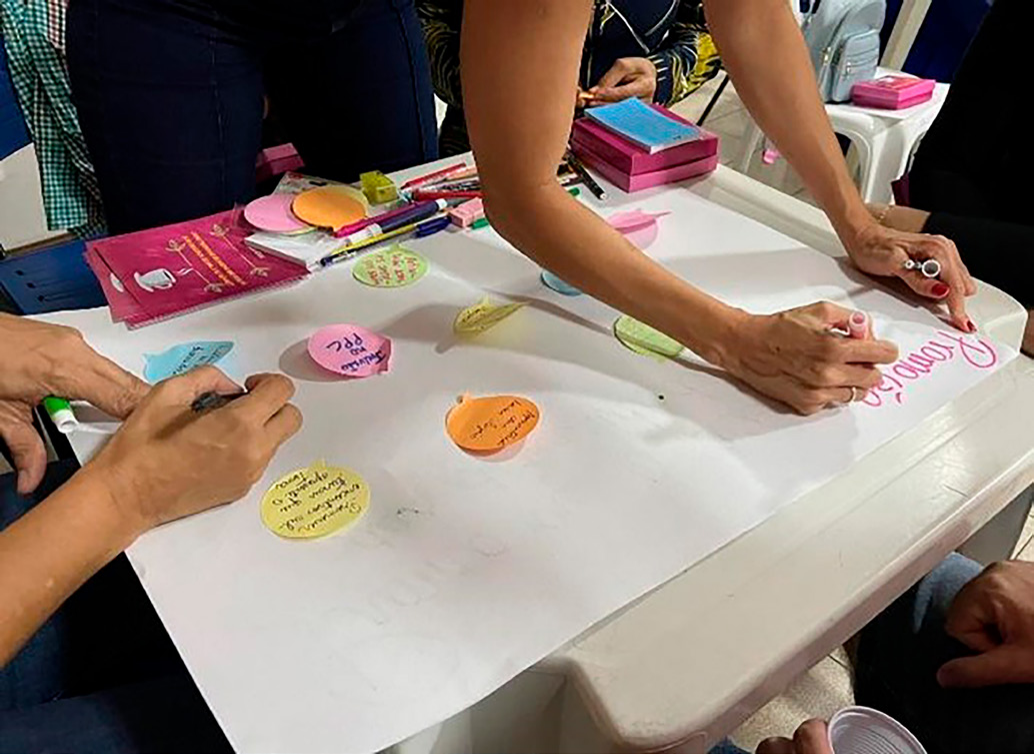
Production of insights- World Café- Belém, Pará-Brazil, 2023.

Participants actively discussed the topic at hand and recorded observations that enriched the groups’ reflections. Furthermore, they produced materials using stationery with the purpose of sharing them among the groups (Figures 3 and 4).

After analyzing the audios obtained during the World Café, the participants’ statements were thoroughly examined, and thematic categories were identified to present the results.

### LGBTQIA+ Education, Training and Health

The analysis of the scores of the world café participants showed that the training of professionals for the behavior adopted during consultations is essential, as its reflection on the therapeutic approach is revealed as a relevant aspect for understanding the dynamics of care, as the following statements point out: […] *When watching the video we realize that the training of professionals, and the behavior of a professional who welcomes the person who comes for their consultation, is decisive in the way the treatment and therapeutic condition are conducted... It is decisive in the sense that I can deny the existence of a person or I can welcome and recognize that person’s real needs* (01).

In the subsequent reflection, participants address the need to change the Course Political Project (PPC), highlighting the importance of making it dynamic. It is understood that to make substantial changes, culture has to be transformed and the training processes reformulated throughout the entire university experience. One of the reports portrayed this need: […] *change the Political Pedagogical Project that has to be alive, we have talked about this, but our discussion does not guarantee that we will have an education that promotes the acceptance of sexual diversity and gender identity, which is why we need to make a cultural change as well, a change in training* (01).

The reports emphasize that merely reviewing this project does not ensure an educational environment that respects sexual diversity and gender identity. This practice must be considered as an ongoing construction, shaped by history and culture. One of the professors highlighted: […] *We know students’ habits when they encounter this diversity, they are transformed, so we need to promote this not only in specific moments in practice, but at all times at the university as a historical and cultural construction* (01).

Professors address the topic of permanent education, emphasizing the need for inclusion, not just as an isolated curricular activity, but as a transversal element of the PPC. They also emphasize that sexual and gender diversity must be comprehensively integrated into the contexts of adult, older people, child and adolescent’s health, seeking an inclusive education that is sensitive to the different dimensions of health, as indicated in the following statement: […] *We talk about permanent education and inclusion, even in the PPC, not just as a curricular activity, but sexual and gender diversity needs to be transversally included in adult’s health, in the older people’s health, in children and adolescents’ health* (01).

It is understood that the connection between training and health promotion is vital for inclusive education, thus emphasizing the need to reformulate the way of teaching to achieve health promotion. According to the reports analyzed, the importance of adjusting the Political Pedagogical Project is understood, integrating inclusion throughout the university experience.

### From Prejudice to the Recognition of Rights

In the professors’ statements, it is clear that equity in the educational environment is important; however, it is important to seek a deeper understanding of its true meaning. This way, the importance of critical reflection for effective changes in the educational scenario is emphasized, as described in the following statements: […] *we talk about equity, but when we have the opportunity to develop equity with one student and the other, we don’t do it and we deny equity in practice, then we need to work on the issue of equity* (02). […] *We can transform ourselves and for that to happen, for this change to happen we need to discuss concepts and prejudices. So we need to discuss concepts of what equity really is* (01).

It stands to reason that there is a need to address and understand how prejudices are configured in society and how they influence health: […] *I think that we need to work on prejudices, what is prejudice, how it is constituted, how it is formed* (01). Thus, the groups draw attention to the importance of understanding the modus operandi of social determinants in health, reflecting on the political process involved in this context: […] *deconstructing from this perspective of society means bringing this process to the legal political framework so that this person feels like they belong in this society, so that wherever they go, they see themselves in this process* (03).

The importance of legislation that protects the rights of LGBTQIA+ people is clear, given the prevalence of prejudice and discrimination they face simply for being who they are: [...] *We discussed it in the second group, in fact, we spoke first, but we need to present the laws, the legal bases and make it clear that the attitudes taken are not just my way, I don’t need to change because it’s my way, I need to change because it’s a law that guarantees someone’s rights* (01).

An example of this is the report that establishes a correlation between personal aversions and the obligation to offer adequate care. However, the quality of health care should not depend on personal acceptance, but rather on meeting the person’s health needs, regardless of their identity, race or sexual orientation: […] *it’s that thing... even if you don’t like black people, gays, you have to treat them well...* (03).

In the reports presented, the need to defend human rights is highlighted. Furthermore, it is important to understand laws and legal principles as essential foundations for promoting health, emphasizing that decision-making should not be just a matter of personal preference. This point is described in the following account: […] *professionals also need to understand this new demand, which is not new, but which has to be understood and met as a demand with rights. And then we culturally construct the concept, because culture takes time, you go through a process, but as a law I can even be xenophobic, but I cannot act in a xenophobic way, I think this is an issue that we need to discuss in college* (01).

Moreover, the importance of incorporating diversity into the healthcare field is underlined, training professionals to adequately meet the health-related demands of the LGBTQIA+ population. The reports presented underscore the relevance of equity in the educational environment, placing emphasis on the disparity between theory and practice that demands critical reflection and effective action to promote real changes, that is, it is not enough to just talk about equity, it is necessary to implement it in practice.

### Reflective Education in the Recognition and Deconstruction of Social Determinants

In the dialogues involving the participating groups, a reflection emerges on the need for reflective political education that aims to deconstruct prejudices. Initially, one of the professors highlights the importance of getting rid of prejudices to think about this reflective political education. In turn, another professor gives prominence to the need for knowledge, understanding, and information as essential for education.

During the participants’ interaction, they broaden the discussion, considering the deconstruction of prejudices a complex challenge in the face of structural issues such as racism, sexism, and LGBTQIA+phobia: […] *about this deconstruction in relation to racism, sexism, LGBTphobia, etc., these are structural issues... they are extremely complex phenomena and they are there as a social structure* (02). […] *we first think about the importance of knowledge, of understanding, of information, because without information and without knowledge we cannot deconstruct ourselves* (04).

Furthermore, the concept of integrality is noted not only in the context of care, but also in the interaction between the university and other institutional services, highlighting the relevance of this integration for the quality of care: […] *we initially thought that to think about this reflective political education we need to deconstruct ourselves and get rid of prejudice* (02).

The importance of understanding LGBTQIA+phobia as a social health determinant is clear, being a marker related to mental health, as mentioned in another report: […] *there was this construction with the acronym LGBTphobia which is a determinant, right? We have experiences that people come to consultations, especially when they are trans women or men, the issue of LGBTphobia is a marker regarding their mental health and other issues* (04).

From this perspective, LGBTQIA+phobia is perceived as a factor that significantly impacts health. Therefore, the dissemination of this knowledge becomes essential for people to understand LGBTQIA+phobia as a SDH: […] *when I think about the incidence and prevalence of diseases, among other things, they are statistically higher when I compare them with the population. So, it is in fact a determination, a social determinant, but many people don’t realize it, so we needed to spread this knowledge so that people realize that it is a determinant* (05).

In the reports presented, the emphasis is on the need to understand and deconstruct the concepts that make up the acronym LGBTQIA+. Questions about the meaning of “I” (Intersex), “T” (Transsexual), “N” (Non-binary) and “+” highlight the importance of internalizing these terms to recognize them as determinants in the experience of these identities: […] *I wanted to know what I is, what T is, what N is, what this + is, because if I don’t even really know the concept, how am I going to think that it is a determinant, and that it is something new, so we need to understand these concepts* (04).

In addition, knowledge is presented as a crucial tool for the integration of the political-legal framework, so that when faced with discriminatory situations, people can see themselves as active participants in the transformation process: […] *deconstructing from this perspective of society means bringing this process to the legal political framework so that this person feels like they belong in this society so that wherever they go, they see themselves in this process. We relate the fact of calling a football player a monkey and today we say that this is racism and that this cannot happen, this is not freedom of expression... this is called something else, so for these people we need this legal political framework* (04).

During the presentations, the importance of addressing integrality in the health area was also highlighted, especially in the context of women. There is a critical reflection on the traditional conception, which often limits integrality to the corporeal aspect. However, participants emphasize the need to overcome this approach and incorporate the dimension of full life: […] *I think we also need to be careful with this integrality thing, because in the health area integrality has a lot to do with mentioning the body and the mind, but I think we need to talk about full life because sometimes the fact of attending to a person and not asking, for example, about their sexual orientation* (01) […]. *We work a lot on this issue of the integrality of women... there is still a constructed concept... even though we already bring up the holistic issue, but it is still constructed on the concept of the corporeal* (02).

Professors emphasize the importance of considering people’s full lives, avoiding stereotypes that automatically associate motherhood with heterosexuality, as evidenced in an example from the video discussed: […] *in the case of the film, she told me that she has two children and then I look and think that she is automatically a mother and that she gave birth because in my social construction, if I have a child, I am straight and I give birth* (05).

In summary, in the World Café dialogues, participants evidence the importance of reflective political education and the deconstruction of prejudices. The discussion addresses the need to understand structural issues related to social markers that can lead to illness. Briefly, it is clear that participants recognize the relevance of education, deconstruction, integration, and knowledge for an inclusive and healthy society.

## DISCUSSION

In this study, central concepts of Foucaultian thought were analyzed, including knowledge and power, which are described in the results of this article as significant markers in teaching practice. This approach provides a meaningful debate about gender and sexuality.

It is important to note that knowledge is not limited to a mere accumulation of information on a specific topic; it goes beyond that, it is considered a tangle of historical and social constructs that impact communication, social interactions, and even the field of education^(20)^.

Through the analysis of the statements, the need for change is noted, but it is not limited to simply modifying institutional policies and curricula; it is, in fact, about transforming the power relations and discourses that support educational conventions. This demands critical reflection on the social and political norms associated with the teaching process^(20,21)^.

The statements presented in the results were relevant regarding the importance of cultural change in training, but for this to happen it is important to recognize the entire historical, political, and social structure related to rights violations that affect the LGBTQIA+ population.

Sexuality is part of social and historical constructions, being seen as a field where power relations are manifested through “sexuality devices”. These devices encompass discourses, norms and practices that shape and define “truths” about subjects, often naturalizing knowledge^(20)^.

It is understood that the training process goes beyond a simple curricular change, but a reconfiguration of knowledge-power relations within institutional education spaces. Therefore, changes in the training process are not just a matter of including new content, but a transformation in the relationships that can shape education^(22)^.

Recognition of the LGBTQIA+ acronym and policies aimed at assisting this group can contribute to better quality of care; furthermore, it makes nursing students feel more qualified to provide equal and prejudice-free care^(23)^.

Identifying and understanding these sexual orientations and identities are essential for care planning. Research participants highlighted this need, emphasizing the importance of implementing structural changes in the educational curriculum.

The participants of the World Café also expressed the necessity of a cultural change in teaching, as many markers of stigma and prejudice have their roots in historical events; for example, in 1870, homosexuality began to be studied and analyzed, undergoing medical interventions and interactions with the aim of “curing” what was called “sick of the sexual instinct”. These individuals were considered abnormal and carnal, evidencing the influence of historical events in the formation of stigmas and prejudices^(20)^.

This way, it is understood that the curriculum and the PPC are not merely technical documents, but rather power devices that shape teaching and discourses within the university. They reflect dominant social and political norms, and influence the way sexual and gender diversity is addressed in education^(20,21)^.

The analysis of the results indicates the importance of understanding discursive practices in the training process, considering prejudice and discrimination as social determinants that affect health promotion^(3)^; however, the inclusion of these themes cannot be seen simply as a content update, but rather a transformation in the way this knowledge is generated and transmitted.

The curricular change that respects human rights was cited as important in this educational process, as it will serve as a guide for inclusive conduct in the teaching environment. This will facilitate the development of educational practices that respect diversity.

Therefore, educational institutions need to critically discuss issues related to discrimination and prejudice, as they are considered power relations in society and human rights violations. Therefore, including topics related to sexuality and sexual diversity in the training of future health professionals is vital, as, in addition to contributing with technical knowledge, it also challenges social and institutional standards that perpetuate discrimination and exclusion^(24)^.

Foucault highlights that sexual repression is deeply rooted, opening space for debate on: power, knowledge, and pleasure, as well as for discourse on human sexuality. This includes formulations, prohibitions, permissions and even relationships linked to institutions^(20)^.

Therefore, nursing curricula has to cover the changes in society, not being restricted to the standards that determine what is described as socially normal, aiming to train professionals who are more qualified to deal with sexual and gender diversity. In this sense, the relevance of transversality in curricular content is highlighted, through comprehensive and equitable care in the application of health policies^(5)^.

It is worth noting that values, beliefs, and prejudices are present in institutional educational settings, as these spaces are not immune to social standards. “These values are historically generated in the cultural contexts of society and, because they are emotionally rooted, they resist change”^(25)^.

It is understood that the lack of knowledge about the health of LGBTQIA+ people contributes to unqualified assistance. Therefore, it is important to note that, from their academic training, students are committed to the principles and policies of health promotion^(1)^.

The historical framework related to knowledge exerts a significant influence on education, as institutions contribute to the dissemination of power in their relationships. Therefore, it is essential to understand the care provided, especially in the context of nursing training^(26)^.

In these terms, some aspects were observed in the results, among them the difficulty of some professors to understand concepts related to the LGBTQIA+ population. This is because heterosexuality and cisgenderism are considered legitimate norms. Normative conceptions of heteronormativity and gender binarism show that these norms can limit knowledge and understanding of diversity^(1)^.

Heteronormativity is linked to power and knowledge, as they influence teaching practices. Foucault analyzes how the influence of knowledge is presented to society, understanding the valorization, distribution, division and attribution of knowledge in pedagogical relations in the institutional environment^(20)^.

Cisheteronormativity refers to a set of power relations that normalize sexuality, gender and sex, based on the idea that only heterosexuality and gender binarism are natural. This view imposes a single standard of sexual orientation and gender identity as acceptable^(27)^.

Those who deviate from the heteronormative standard often face processes of inferiorization, resulting in a reduction in self-esteem and, consequently, in both physical and mental complications. Furthermore, it is wrong to say that these cases are individual and not collective, especially when they occur in institutional environments such as universities^(28)^.

Thus, it is understood that cultural change is a prominent element in the training of health professionals, as the provision of health care with a culturally sensitive approach becomes viable when it is intrinsically linked to the recognition of cultural diversity and the understanding of how this diversity impacts the process of seeking care. Furthermore, this type of care criticizes the biomedical model and favors social transformations related to issues involving social justice and human rights^(29)^.

In this sense, it is imperative that, since the nursing training phase, skills aimed at promoting health are developed, taking into account issues of violence, gender, and sexuality as factors related to illness^(1)^.

Therefore, a careful analysis of nursing course curricula should be carried out in light of social changes, as they are still based on heterocisnormative standards, providing students with little preparation to deal with the specific needs of this portion of the population. Thus, an integrated approach to these contents is essential in curricula in all areas of direct care for human beings, such as child health, adolescent health, women’s health, adult’s health, and older people’s health^(5)^.

Consequently, it is understood that deconstruction on the education field should not be limited to the individual sphere, but rather occur collectively. It is necessary to promote changes in institutional structures and discourses. This involves not only raising awareness about injustices and inequalities, but also mobilizing political and institutional actions to confront them.

The clarification and discussion carried out are fundamental to understand nursing teaching practices and, thus, adds to reflection on nursing teaching methodologies and their contributions to public health.

As a limitation of the study, the number of participants stands out, given the heterogeneity of the institution’s teaching staff. Furthermore, it is understood that as this is a sensitive discussion from a historical and social perspective, the reports may not fully correspond to the reality presented. However, a discussion was held with several authors to address the gaps presented from the perspective of dialogue.

## CONCLUSION

Reshaping the way health care is taught and practiced is critical to inclusive and equitable education. Therefore, it is necessary for academic curricula to integrate themes involving sexual and gender diversity into their content, seeking to deconstruct prejudices through reflective political education.

The professors participating in the research recognize the importance of a cultural change to promote the health of the LGBTQIA+ population. However, they signal the need to deconstruct historically rooted concepts.

The importance of recognizing the social determinants of health that affect the LGBTQIA+ population is highlighted, to promote health care that meets the needs of this group. Nevertheless, to achieve this goal, critical reflection is required to overcome existing disparities in the health field.

The results also highlight the importance of deconstructing prejudices, which is a key factor in teaching practices that comprise social health needs, promoting a healthier and more inclusive environment.

Furthermore, this study highlights the relevance of including Foucaultian concepts for a reflection on knowledge in educational practice. The analysis revealed that these terms are necessary in promoting debate on gender, sexuality and education, as they influence education.
